# Confining Domains Lead to Reaction Bursts: Reaction Kinetics in the Plasma Membrane

**DOI:** 10.1371/journal.pone.0032948

**Published:** 2012-03-27

**Authors:** Ziya Kalay, Takahiro K. Fujiwara, Akihiro Kusumi

**Affiliations:** 1 Institute for Integrated Cell-Material Sciences (WPI-iCeMS), Kyoto University, Kyoto, Japan; 2 Institute for Frontier Medical Sciences, Kyoto University, Kyoto, Japan; Centrum Wiskunde & Informatica (CWI) & Netherlands Institute for Systems Biology, The Netherlands

## Abstract

Confinement of molecules in specific small volumes and areas within a cell is likely to be a general strategy that is developed during evolution for regulating the interactions and functions of biomolecules. The cellular plasma membrane, which is the outermost membrane that surrounds the entire cell, was considered to be a continuous two-dimensional liquid, but it is becoming clear that it consists of numerous nano-meso-scale domains with various lifetimes, such as raft domains and cytoskeleton-induced compartments, and membrane molecules are dynamically trapped in these domains. In this article, we give a theoretical account on the effects of molecular confinement on reversible bimolecular reactions in a partitioned surface such as the plasma membrane. By performing simulations based on a lattice-based model of diffusion and reaction, we found that in the presence of membrane partitioning, bimolecular reactions that occur in each compartment proceed in bursts during which the reaction rate is sharply and briefly increased even though the asymptotic reaction rate remains the same. We characterized the time between reaction bursts and the burst amplitude as a function of the model parameters, and discussed the biological significance of the reaction bursts in the presence of strong inhibitor activity.

## Introduction

Reaction kinetics is undoubtedly one of the most immensely studied subjects in chemistry and physics, both from theoretical and experimental aspects. Until recently, the majority of the studies in this field was focused on obtaining quantities relevant in the thermodynamic limit such as bulk reaction rates. Nevertheless, some of the salient features of chemical reactions in complex systems such as a cell are the large fluctuation in the number density of reactants [1], [2], the presence of mesoscale structures that can confine reactants [3] and alter reaction rates [4]–[6], and molecular crowding induced effects due to the presence a high concentration of inert molecules [7]–[9].

The cellular plasma membrane is considered to be a two-dimensional liquid [10], where many membrane molecules perform various vital functions for the cell, including signal processing and selective internalization of external molecules. Many of these functions are made possible due to the bimolecular reactions of specific membrane molecules within the two-dimensional liquid space of the plasma membrane. However, the plasma membrane is far from an ideal two-dimensional solution. In addition to specialized membrane regions, such as cell-cell and cell-substrate adhesion structures, clathrin-coated pits, and caveolae, even the general membrane area consists of nano-meso-scale domains [11]. Especially two kinds of membrane domains, raft domains [12] and domains induced by the actin cytoskeleton (compartments) [13], where membrane molecules are temporarily confined, are considered to be two organizing principles of the plasma membrane. For instance, recent experimental and computational findings provided evidence for how molecular confinement in cytoskeleton induced domains can affect the dynamics of EGF (epidermal growth factor) [14]–[16] and B-cell receptor signaling [17], how lipid rafts can enhance the interaction between receptor proteins and their downstream signaling molecules [18], [19], and how membrane molecules are brought closer to each other by active processes involving actin filaments [20], [21].

The purpose of this article is to address the implication of plasma membrane compartmentalization on reversible bimolecular reactions. In many computational studies it was found that confinement may or may not enhance reaction rates and modify the local distribution of reactants [4], [22], [23]. For instance, Nicolau et al. [4] demonstrated that raft like domains can increase the local concentration of proteins, which in turn enhances the local reaction rate if the raft size is small enough. Despite these findings, a clear quantitative result that relates the strength of confinement effect in a surface partitioned into compartments to the statistics of reaction events has been missing. Our study is also intended to fill this gap by considering a simple model that captures the essential aspects of confining domains of this type.

**Figure 1:**
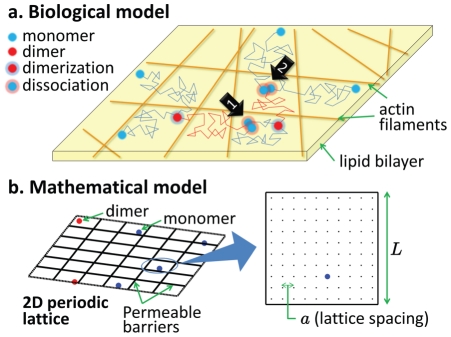
The models of reversible dimer formation in the compartmentalized plasma membrane. **a.** Schematic illustration of the compartmentalized view of the plasma membrane according to the membrane skeleton fence model [13], and the kinetics of reversible dimer formation as described in the text. The thin lines correspond to the random paths traced by the molecules while diffusing in the membrane. **b.** illustration of the lattice-based model of diffusion and reaction in two-dimensions (2D) (see the *Methods* section for details, and Video S1 for an animation based on this model).

Single molecule/particle tracking experiments performed at ultrafast frame rates showed that transmembrane proteins and phospholipids are temporarily confined in mesoscopic domains of size 30–250 nm, for a typical duration of 1–100 ms in many cell types [24], [25]. According to the membrane skeleton fence model [13], these domains, which we will call *compartments*, are induced by a meshwork of actin filaments lying in close proximity of the plasma membrane, as imaged by electron tomography technique [26]. The actin-induced membrane compartmentalization in the cellular plasma membrane is an extremely important field in cell biology, represented by more than 2,000 publications [27], [28], and has strong impact on signal transduction and molecular trafficking in the cell membrane. Membrane molecules can get temporarily confined in these compartments which hinders their diffusion at a time scale longer than the typical confinement, or escape time. As a result, a molecule appears to have a larger diffusion coefficient 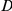 when observed at time scales shorter than the escape time, and an effective diffusion coefficient 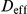 when observed for much longer periods. The ratio 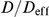 was found to lie between 5 to 50 in different cell types (see Table 1 in ref. [25]). We consider the effects of these compartments on the kinetics of reversible dimerization as illustrated in Figure 1(a). Due to temporary confinement, dissociating molecules can quickly recombine if they stay in the same compartment for long enough, or they can perform a long excursion without encountering each other, as exemplified by the trajectories shown by arrows (1) and (2) in Figure 1(a).

Microscopic models employed to investigate reaction kinetics can be roughly categorized into two: those that treat reactants as randomly moving molecules in continuum, and those in which reactants can only occupy discrete points in space, i.e. lattice-based models. Models from both categories have met with different levels of success in reproducing the observed chemical kinetics. For a review on modeling reaction kinetics in biological membranes see Melo and Martins [29], and for a recent discussion on bridging different kinds of models, see Fange et al. [30]. In this article, we present a theoretical analysis of reaction kinetics in the plasma membrane viewed as a compartmentalized surface, where the compartments are confining domains induced by the actin cytoskeleton, as illustrated in Figure 1(a). Using kinetic Monte Carlo simulations, we study the dynamic equilibrium between monomers and dimers that perform a discrete time random walk in a two-dimensional lattice partitioned into compartments by periodically placed permeable barriers. We note that the results obtained by lattice based models may differ from those of continuum models [29], [31]; however, in this work we are only interested in comparing the reaction kinetics in the presence and absence of compartments, and not in predicting absolute values for reaction rates. The details of the model are described in the *Methods* section, and the full derivations of the theoretical results can be found in the Text S1. In the next section, we report the results of our Monte Carlo simulations which demonstrate that confining domains can significantly change the temporal pattern of biochemical reactions by inducing *reaction bursts*.

## Results

### Confinement strength and escape time distribution

Confinement effects on reaction kinetics can be strong or negligible depending on the parameters of the system, that are: the microscopic diffusion coefficient 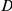, characteristic size of confinement 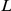, and the permeability of the boundaries of a confining domain. If the confinement effect is strong, a diffusing molecule would be evenly distributed over a confining domain before it escapes. If a molecule is diffusing with a diffusion coefficient 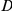 in two-dimensions, the typical time it takes for it to cover an area 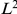 is given by 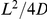. Similarly, the time it takes for a diffusing molecule to escape from a square region of area 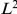 with permeable walls can be well approximated by 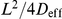 where 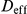 is the effective, *long time*, diffusion coefficient that is a function of 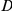, 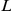, and the permeability of domain boundaries. In some simple cases where the barriers are periodically placed, 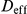 can be calculated exactly [32], [33]. Therefore, when the confinement effect is strong, we expect
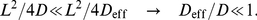
(1)It is straightforward to show that for a molecule diffusing in a square region of area 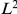 with permeable boundaries, the ratio 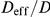 is given by (see Text S1 and Figure S1)
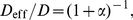
(2)where 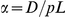, and 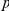 is a quantity (non-negative) with the dimensions of lenght/time that characterizes the permeability of domain boundaries. For a completely confining domain, 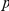 is equal to 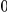; whereas, in the absence of confinement, it goes to infinity. As shown in the Text S1, 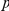 is related to the other key quantities through 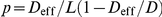. The dimensionless parameter 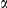 characterizes the strength of confinement and confinement effects will be pronounced when 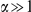. In the Text S1, we show that the distribution of escape times for a random walker trapped in a square region of area 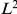, averaged over all initial positions, can be written as a sum of exponentials
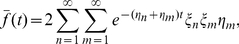
(3)where 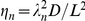, 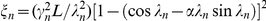, 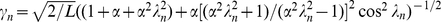, and 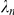
's are the positive solutions of 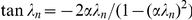. If 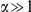, the distribution of escape times, even when the particle starts near the compartment boundary, can be well approximated by a single exponential distribution (see Text S1).

### Simulation results

**Figure 2:**
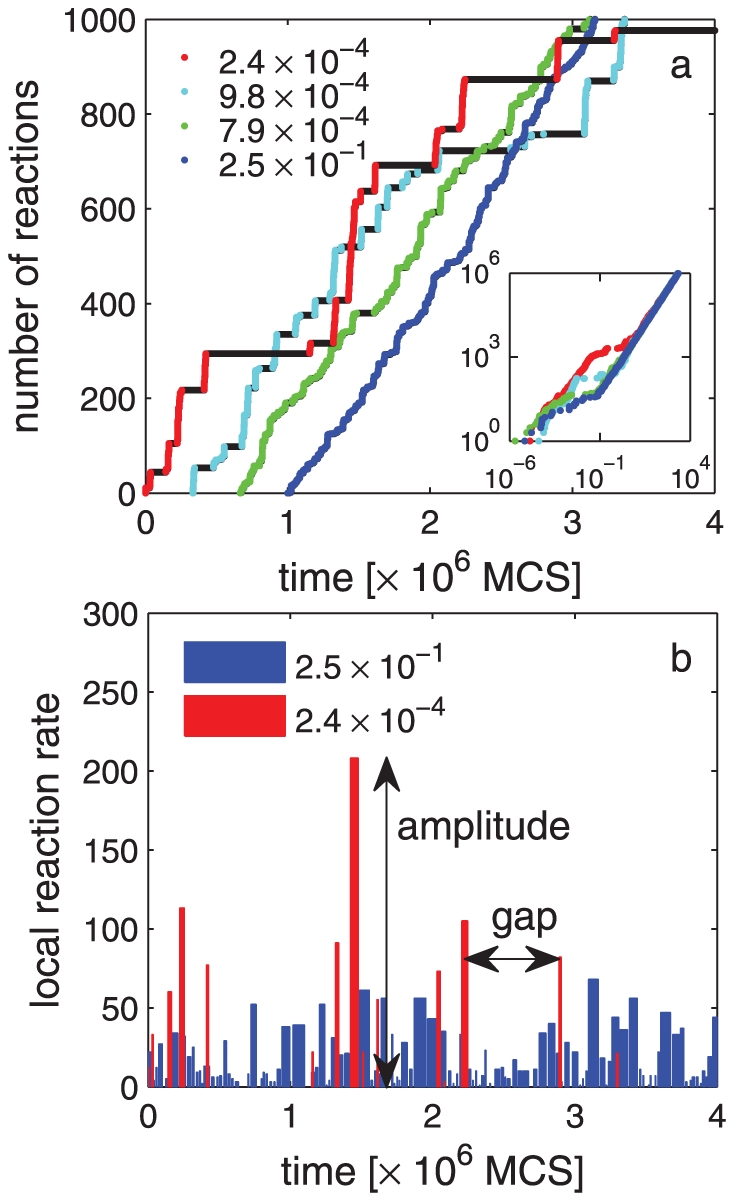
Total number of reactions, and local reaction rate as a function of time. **a.** Total number of reactions involving the tracer molecule as a function of time, obtained by Monte Carlo simulations. In all plots, black data points correspond to no activity and colored data points indicate reaction events. Different colors correspond to different confinement strengths such that 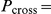 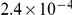
(red), 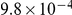
(cyan), 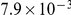
(green), and 0.25(blue) which represents the case of no confinement. The values of other parameters are: 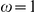, i.e. reactions are diffusion limited, 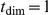, 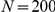, 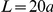, and there are 10 molecules in the lattice such that 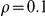. The main figure shows short samples of the simulation data, covering 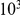 reaction events for each case, and is plotted with an arbitrary offset for visual clarity. The inset shows full data sets for single runs on logarithmic axes. **b.** local reaction rate as a function of time for strong confinement 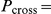 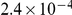
(red), and for no confinement 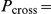
0.25(blue). All parameter values are the same as in **a.** When confinement is strong, the local reaction rate exhibits *bursts* during which its *amplitude* is abruptly increased, that are followed by silent periods, or *gaps*. See the text for the details of how local rate is calculated. In **b**, 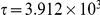 simulation steps.

To monitor the reaction kinetics, we labeled one of the molecules in the simulation region as the *tracer*, and kept following it in time. Whenever the tracer formed a dimer with another molecule, we recorded this as a reaction event, and when this dimer dissociated, we recorded it as a dissociation event, and continued to follow the tracer. Therefore, the reaction rate we are interested in calculating is not the overall rate of dimerization in the whole system; it is rather the reaction rate at the single molecule level. In order to quantify changes in reaction rate, we determined the periods over which the time between subsequent reactions does not exceed a certain value 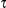, which we refer to as *bursts* (see Figure 2(a,b)). In the presence of confining domains, there exists a characteristic time that can be used in choosing 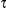, which is the time it takes for one of the particles to escape from a compartment, as explained in detail in the *Methods* section (see Eq. (10)). Setting 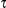 equal to the escape time makes it likely that the subsequent interactions between two particles that dissociate at 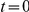 and react again before one of them leaves the compartment are assigned to the same *reaction burst*. In our study, depending on the confinement strength, the value of 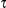 ranges between 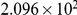 and 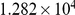 simulation steps. We termed the time between two subsequent bursts a *gap*, and the total number of reactions during a burst, the burst *amplitude*, as illustrated in Figure 2(b). Therefore, the burst amplitude can be interpreted as the *local reaction rate*. To facilitate understanding, an animation based on our simulation that visualizes reaction kinetics and bursts is available as a Video S1.

In all simulation runs, the simulation region was taken to be a square lattice of 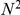 sites, and each compartment to be a square of 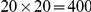 lattice sites such that 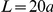, where 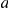 is the distance between adjacent lattice sites. When a particle is adjacent to a compartment boundary, it crosses into the adjacent compartment with probability 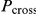, and takes steps in other directions (within the compartment) with equal probability. In accordance with experimental findings on molecular diffusion in plasma membranes [25], if we consider 40 nm compartments and a diffusion coefficient 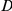 of 8 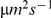 (within a compartment), resulting in 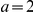 nm, a simulation time step will correspond to 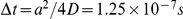. During every run of the simulations, the typical number of time steps, Monte Carlo steps (MCS), was on the order of 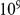 such that the system evolved for more than tens of seconds. We frequently considered the value 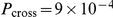 that corresponds to 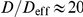 (or 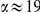), which is typical for the molecules in the plasma membrane of live cells [13].

**Figure 3:**
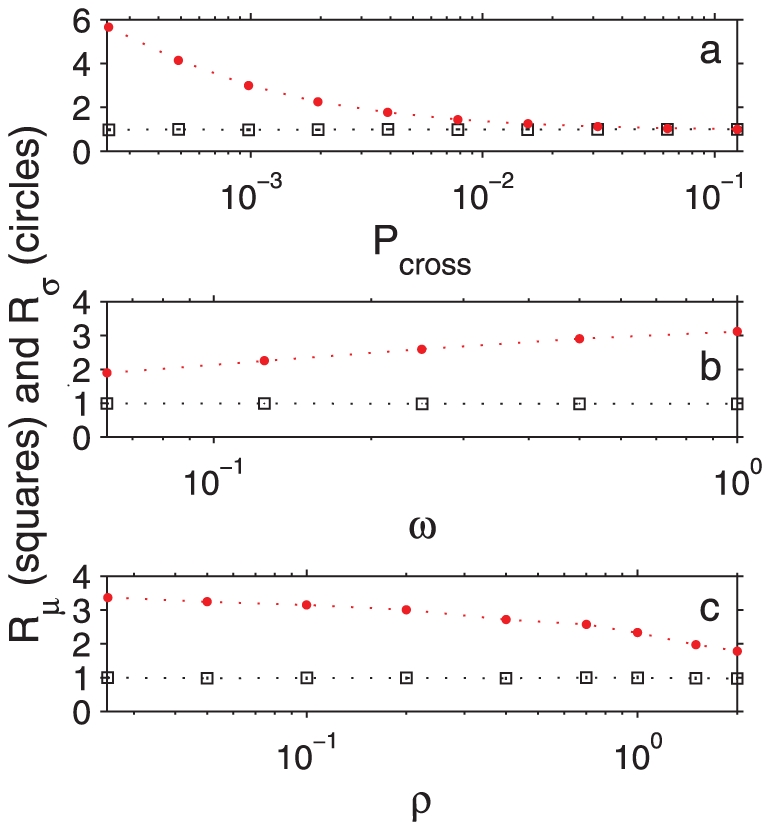
Behavior of the ratio of the mean and standard deviation of the time between reactions in the presence of confining domains to that in the absence of confining domains, denoted by 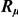
 **and** 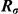 **, respectively (see text).** Data obtained by Monte Carlo simulations. **a.** the dependence of 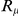 and 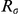 on confinement strength characterized by 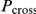. 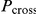 takes on values between 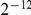 and 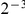. Other parameter values are: 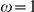, 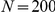, 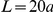, 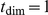, 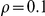. **b.** 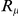 and 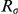 as a function of reaction probability 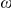, with other parameters fixed at: 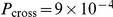, 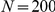, 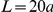, 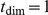, 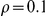. **c.** density dependence of 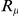 and 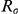. In this case 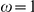, 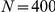, 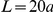, 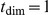, and the density 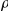 varies between 0.05 and 2 molecules per compartment. Error bars in all plots are smaller than the data points.

**Figure 4:**
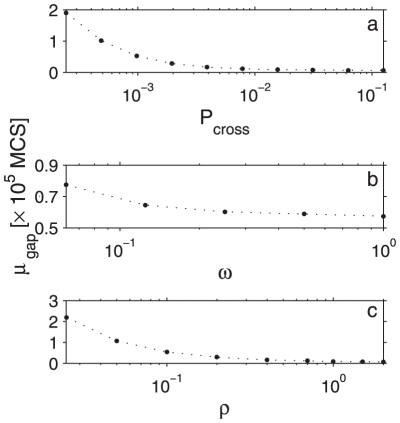
The mean gap duration, or the time between subsequent bursts, as a function of model parameters, obtained by Monte Carlo simulations. **a.** the effect of confinement strength on 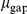. 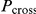 varies between 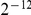 and 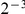. Other parameter values are: 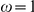, 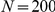, 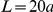, 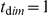, 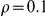. In order to identify bursts, we considered 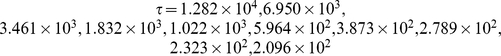, for 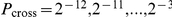, respectively. **b.** 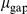 plotted against 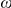, with other parameters fixed at: 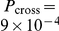, 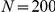, 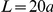, 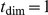, 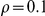. **c.** 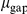 as a function of density. Parameters were fixed at: 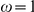, 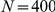, 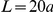, 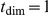, and 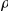 varies between 0.05 and 2 molecules per compartment. Error bars in all plots are smaller than the data points. In **b** and **c**, 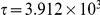 simulation steps.

**Figure 5:**
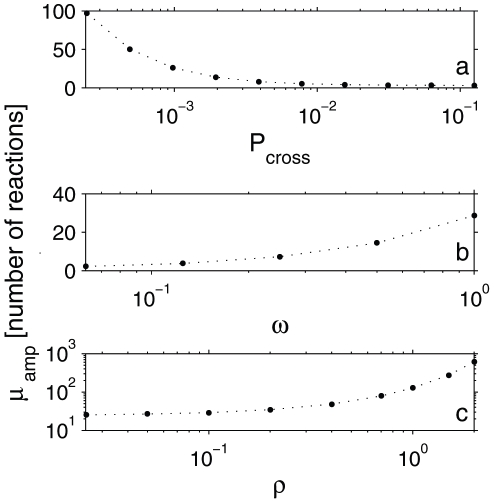
The mean burst amplitude versus model parameters, obtained by Monte Carlo simulations. **a.** the effect of confinement strength on 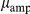. 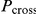 varies between 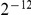 and 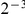. Other parameter values are: 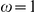, 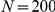, 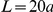, 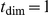, 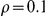. In order to identify bursts, we considered 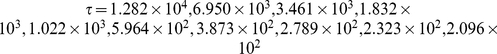, for 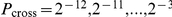, respectively. **b.** 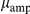 as a function of 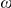, with other parameters fixed at: 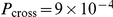, 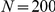, 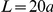, 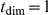, 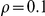. **c.** 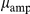 as a function of density. Parameters were fixed at: 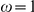, 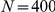, 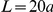, 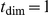, and 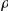 varies between 0.05 and 2 molecules per compartment. Error bars in all plots are smaller than the data points. In **b** and **c**, 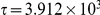 simulation steps.

As explained below, our main results are displayed in Figures 2, 3, 4, and 5, and demonstrate the influence of confinement strength, activation energy, and the number density of molecules on reaction kinetics. The effect of dimer lifetime, denoted by 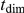, was found to be weak, and is only discussed in the Text S1 and demonstrated in Figure S2.

#### Confinement strength

As the compartment boundaries become less and less permeable, reaction bursts become more and more apparent. In order to study this effect quantitatively, we varied 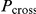 between 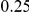 (no confinement) and 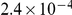 (strong confinement) and calculated the time dependence of the total number of reactions, and the local reaction rate.

We systematically investigated the effect of confinement strength on the mean and variance of the time between reactions (Figure 3(a)). For a brief discussion on the full distribution of time between reactions, see Text S1 and Figure S4. In displaying our findings, we found it convenient to define 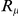 and 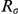 as ratios of the mean and standard deviation of the time between two subsequent reactions in the presence of confining domains (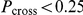) to that in free space (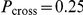). As seen in Figure 3(a), 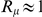 for all confinement strengths but 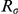 differs significantly from 1 as confinement gets stronger. Therefore, our simulations indicated that the asymptotic mean reaction rate does not depend on the confinement strength, as shown in the inset of Figure 2(a). However, the variance of the time between reactions was found to depend strongly on the confinement strength (Figure 3(a)). A theoretical discussion on the invariance of 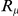 will be given towards the end of this section.

Even though the mean time between reactions did not depend on confinement strength, the same was not true for the mean gap duration, 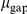, and mean burst amplitude, 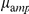, which were both inversely proportional to 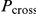 as shown in Figures 4(a) and 5(a).

As a final remark, we should note that the ensemble averaged reaction rate did not depend on confinement strength, as expected (see Text S1 and Figure S3).

#### Activation energy

Most chemical reactions are not expected to take place during the first encounter between molecules as reactants and products are usually separated by an energy barrier. For instance, it has been argued that the fraction of collisions between activated rhodopsin and transducin that lead to photo response lies between 0.1 and 0.01 [34]. In our simulation, we only let two monomers form a dimer with probability 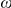 when they encounter. We found that when all other parameters are fixed, the gap duration decreases and the burst amplitude increases with increasing 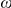, resulting in higher reaction rates. This quite intuitive finding is displayed in Figures 4(b) and 5(b). On the other hand, the ratio 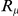 does not depend on 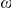 when all other parameters are fixed (Figure 3(b)).

#### Number density of molecules

The rate of encounter between molecules strongly depends on their number density. We denote the number of molecules per compartment as 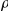, which varied between 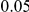 and 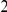 in our simulations. Figures 4(c) and 5(c) show that the gap duration and the burst amplitude also changed significantly with density, such that the overall reaction rate increased with increasing density. However, 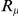 did not depend on the density as shown in Figure 3(c), indicating that the presence or absence of confining domains did not alter the average time between reactions even at different densities. Nevertheless, we should note that even the maximum density of particles that we considered in this study is quite low (1 particle per 200 lattice sites) so that the system was outside the regime where high-density effects such as molecular crowding can be observed.

### Invariance of the mean reaction rate

Our simulations showed that the average time between reactions does not depend on confinement strength (Figure 3(a)). Here we would like to give some theoretical insight into why this is so. In the absence of confining domains, let 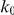 be the reaction rate which is determined by the diffusion coefficient and the number density of molecules alone. In the presence of confining domains, molecular encounters necessarily occur in a hierarchical manner. First, two molecules need to enter the same compartment; second, they need to find each other. If the confinement is strong, the time it takes for two molecules to meet in the same compartment will increase, as they now diffuse with an effective diffusion coefficient 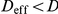. Therefore, the reaction rate 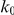 will be rescaled by a factor of 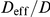. However, molecules that enter the same compartment will encounter many times such that the reaction rate in the presence of confining domains is expressed as
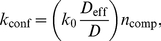
(4)where 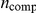 is the mean number of encounters occurred before the molecules shall move into different compartments. Note that 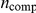 is equal to 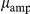 with 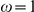 so that we can use Eqs. (9) and (11) (see *Methods*) to deduce
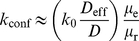
(5)to first order in 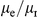, where 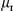 is the average time it takes for the tracer and its last encounter partner to reencounter following a dissociation at 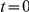 supposing that they can never escape from the compartment, and 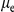 is the average time it takes before one of the particles escape from the compartment. While 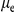 is given by Eq. (10) (see *Methods*), it is far more difficult to obtain an analytical expression for 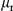. Nevertheless, we expect 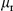 to be a multiple of 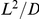, as this is the only timescale involved in the problem of reencounter before the molecules can leave a compartment. Using Eq. (10) and taking 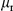 to be the product of an undetermined constant and 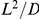, we obtain
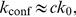
(6)where 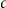 is a constant that does not depend on 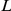, 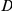 or 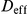. Eq. (6) indicates that in the presence of confining domains, the average reaction rate does not depend on confinement strength and is a multiple of the rate in the absence of compartments, to a first approximation. This is indeed what we found in our simulations, with 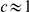, as shown in Figure 3.

To account for the invariance of the mean reaction rate on more general grounds, it is useful to find out how the equilibrium properties of the system are affected by the presence of confining domains. Consider a system of 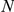 particles undergoing the reaction 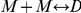 such that the number of monomers (M) and dimers (D) at equilibrium are given by 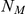 and 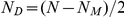, respectively. The energy of this system can be expressed as the sum of the energies of monomers and dimers, 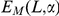 and 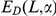, and the interaction energy between them 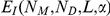. All of these energies may depend on 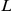, the characteristic length of confinement, 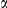, the confinement strength, and other properties of the environment which are not explicitly considered in this discussion. The partition function [35] for this system can be written as
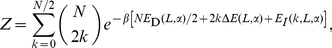
(7)where 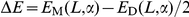, 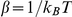, 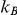 is the Boltzmann constant, 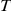 is the absolute temperature, and the index 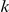 corresponds to the number of pairs of monomers. As we mentioned above, in general, 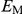, 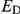 and 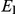 would depend on the properties of the environment where the particles reside, such as the characteristic length of confinement, molecule density, and specific intermolecular interactions. For instance, if one considers significant volume exclusion effects, molecular crowding induced by inert molecules may decrease the energy of the dimer state as the system tries to maximize the free volume [31], so that 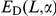 would decrease with decreasing 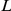. In our system, the characteristic length of confinement is much larger than the size of an individual reactant, the particle density is low (1–20 particle(s) per 4000 lattice sites) such that volume exclusion effects are negligible, and monomers and dimers do not interact to form complexes. In addition, the waiting time distribution is identical for each lattice site including those adjacent to the barriers, both for monomers and dimers, such that the presence of compartments do not alter the equilibrium distribution of particles. Therefore, we can treat 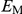 and 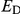 as parameters that do not depend on 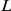, and 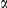, and neglect 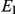. Under these assumptions, the partition function can be approximated for large values of 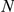, and the fraction of monomers at equilibrium can be written as (see Text S1)
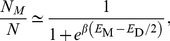
(8)which is equal to 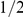 as 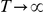, becomes 0 as 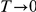 if the dimer state is favorable (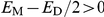), and becomes 1 as 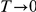 in case the monomer state is favorable (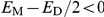). Therefore, in the present case, we argue that the fraction of monomers at equilibrium does not depend on whether confining domains exist or not, and as the reaction probability and dimer lifetime does not depend on the confinement size or strength, it is natural to expect that the average reaction rate will not be modified by the confining domains we consider. However, we should note that the rate at which equilibrium fluctuations decay would depend on the presence of confining domains, causing the significant increase in the variance of the reaction rate induced by confining domains.

## Discussion

In summary, we showed that an array of confining domains such as those observed in the plasma membrane can significantly modify kinetics of reversible reactions by causing each molecule to go through bursts of interactions. We presented detailed results obtained by simulating a lattice-based reaction-diffusion model. We found that the mean time between reactions does not change whether an array of confining domains is present or not, and argued that this is because volume exclusion effects are negligible, and that the presence of compartments do not alter the equilibrium fraction of monomers and dimers. Therefore, even when the diffusion is hindered due to the presence of confining domains, the number of reactions occurred over a sufficiently long time interval does not change, as shown in Figure 2(a)'s inset, and as explained by theoretical arguments in the results section. However, the variance of the time between reactions is significantly different in these two cases, being larger in the presence of confining domains, as shown in Figure 3. This turns out to be an indicator of a profound change in the temporal pattern of reaction events: *bursts of reactions instead of constant but low yield*. Even though the presence of confining domains does not alter the asymptotic or ensemble averaged reaction rates, the spatio-temporal inhomogeneity it introduces can have a significant affect on how the plasma membrane works, as discussed in the rest of this section.

It is well known that reversible phosphorylation, and subsequent activation/deactivation, of proteins via kinases and phosphatases is an important mechanism that regulates many cellular processes [36]. A number of studies employing phosphatase inhibitors showed that phosphatase activity is generally higher than kinase activity to maintain cells in a quiescent state. For example, Klarlund [37] showed that the addition of vanadate, an extensive phosphatase inhibitor, to cultured NRK-1 cells led to a maximal 40-fold increase in phosphotyrosine in the cells without any other extracellular stimulations, and a dose dependent and reversible transformation of phenotype. Lerea et al. [38] found that the treatment of human platelets with vanadate and molybdate, another phosphatase inhibitor, increased the levels of phosphotyrosine and led to the activation of platelets. It is also worthwhile to note that although kinases play an indispensable role in activating molecules, the rate and duration of signaling events may be predominantly determined by phosphatase activity, as suggested by Heinrich et al. [39].

In the light of our findings, we believe that bursts in protein phosphorylation by stimulation-activated kinases within a compartment can locally override phosphatase activity and initiate signaling cascades from this particular compartment (assuming that phosphatases do not exist in every compartment; generally the plasma membrane contains more than a million compartments). This compartment might be the one where the extracellular stimulant molecule was received by a receptor. Thus, the presence of compartments may enable the cell to robustly collect additional information regarding the location of stimulus to be used during processes such as chemotaxis. In the absence of plasma membrane compartmentalization, protein phosphorylation would occur in greater areas but at lower levels, which will be totally washed away by the strong phosphatase activity.

In addition to generating spatial regulation and patterns, plasma membrane compartmentalization might help creating temporal regulation and patterns. If the molecules that go through bursts of reactions induce a set of downstream events, the distribution of these events in time would also follow, or at least be influenced by, the timing of the bursts. Therefore, instead of occurring at a constant or slowly varying rate, the downstream events could proceed in impulses, separated by silent periods. Such *frequency modulation* of events, as opposed to *amplitude modulation*, was indeed observed in yeast cells by Cai et al. [40]. Moreover, in a recent theoretical study on enzymatic reactions, Grima [41] showed that in the presence of submicron compartments and input patterns consisting of bursts, reaction rate can significantly deviate from the predictions of classical reaction kinetics where the discrete nature of reactants is ignored.

Finally, a mechanism that generates reaction bursts can be linked to biological switches [42], [43]. A cell houses an intricate signaling network in which the occurrence of an event triggers the occurrence of many others via molecules that act like messengers. However, local concentration of these messengers fluctuate in time such that they may lead to many false starts which in turn could plague the whole network. Therefore, in intracellular communication, it could be safer to use clear messages in the form of high-amplitude, brief impulses rather than a low amplitude and temporally wide spread signal that can easily be mistaken for noise [18], [19]. In this respect, mesoscopic compartments within the cell, which can induce bursts of molecular interactions as we described in this article, could be more functional than previously thought.

## Methods

### Lattice-based model

We consider 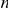 interacting monomers represented by random walkers in a square lattice of 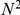 sites with spacing 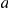 and periodic boundary conditions, partitioned into square compartments of area 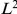 by permeable barriers (see Figure 1(b)). The number density of random walkers per compartment is denoted by 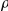. If a monomer or dimer is away from the barriers, at each time step it moves in every 4 directions with equal probability. If it is adjacent to a barrier, it crosses the barrier with probability 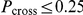 and moves in all other directions with equal chance. When two monomers occupy the same lattice site during a time step, they form a dimer with probability 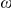 (identical to the probability of reaction during an encounter as defined in the main text) and the dimer dissociates into two monomers after a random time. We considered exponentially distributed dimer lifetimes which are obtained by sampling from an exponential distribution with mean 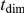, dividing the result by the simulation time step 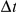 and rounding up to the nearest integer (as we consider a discrete time random walk). Initially all monomers are separated by one lattice site in order to ensure that all of them can encounter each other with our encounter criterion. We focus on the cases where reactant concentration is low such that the interactions between monomers and dimers are negligible, and do not consider these interactions in the simulation. Under physiological conditions, often times the concentration of reactants are low enough so that these assumptions are valid. Typically, there are more than a million compartments in the plasma membrane, whereas the numbers of most molecules that exist in or on the plasma membrane are generally in the range of 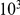
–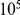 copies per cell [44]. Of course, extreme cases exist such as EGF receptors in cultured A431 cells (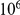
–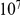 copies per cell).

### Statistics of burst amplitude

Given the tracer reacts with a molecule after entering a compartment, how many more times will it react with the same molecule before they end up in different compartments? This number determines the amplitude of each reaction burst. The number of encounters before an escape, which we denote by 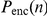, depends on the value of 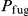, the probability that either one of the molecules will escape the compartment before reencountering the other, where the subscript fug stands for *fugitive*. 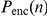 is explicitly given by 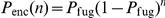. Calculating the probability that 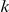 reactions take place after 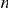 encounters and summing over all possible values of 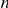 (see Text S1), we obtain the distribution of the number of reactions in a burst, whose mean value is
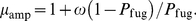
(9)The calculation of 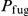 is outlined below.

If the encounter time happens to be larger than the escape time, one of the molecules may leave, leading to a significant increase in the time it takes before the next encounter takes place. Let 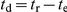 be the random variable that is positive when the tracer leaves the domain before encountering the other molecule and negative otherwise. Here 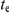 is the time it takes before the any of the two molecules escape from the compartment. Assuming that the escape time can be approximated by a single exponential with mean (see Text S1)
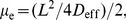
(10)where the factor of two is due to the presence of two molecules, and calculating with what probability 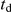 is positive, we obtain
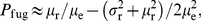
(11)This result is valid when the escape time can be well approximated by a single exponential (see Text S1 for full derivations).

## Supporting Information

Figure S1 **Illustration of the Master equation picture of diffusion with periodically placed barriers.**
(EPS)

Figure S2
**Behavior of** 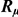 **and** 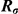 **(described in the text) as a function of** 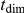 **.** Data obtained by Monte Carlo simulations. Parameters values are 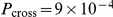, 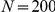, 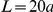, 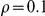. Error bars were obtained by subsampling the data by bootstrapping and indicate 95% confidence intervals.(EPS)

Figure S3 **Total number of reactions involving the tracer molecule as a function of time.** Results are obtained by Monte Carlo simulations (ensemble averaged over 1000 non-overlapping segments of the simulation data). All definitions and parameter values are the same as those of Figure 1(a) of the main text. For visual clarity, each data set is plotted with a different offset along the x-axis.
(EPS)

Figure S4
**Distribution of the time between reactions for different confinement strengths.** **A.** All parameter values are the same as in Fig. 3(a) of the main text. A bin size of 100 simulation steps was used to calculate the probabilities. The numbers in the legend correspond to the value of 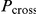. **B.** distribution of the time between reactions for 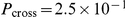 (no confinement) and 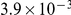, illustrating the qualitative change in the behavior of the curves in different time windows. All parameter values are the same as those in **A**. See text for further details.(EPS)

Text S1 **Supporting information that includes detailed derivations of the mathematical results presented in the main text.**
(PDF)

Video S1
**Visualization of our Monte Carlo simulation based on the lattice model.** Black circles correspond to diffusing reactants that can form a dimer, which is indicated by a red circle. One of the reactants is shown in green and represents the tracer. The 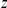-axis is proportional to the local reaction rate, which is the mean number of reactions that took place in the corresponding compartment during the last 100 simulation steps, multiplied by 10. **a.** a simulation run in the absence of compartments (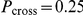). **b.** the effect of compartments on reaction kinetics (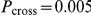). It is readily observed that in **a**, reactions take place more frequently all over the simulation region; however, the local reaction rate, depicted by the height of the bars, is much lower compared to those in **b**. We refer to the brief but large increases in the local reaction rate in **b** as “bursts”, which become more and more apparent with increasing confinement strength. In both cases, the visualization is accelerated such that the configuration of the system is shown only around times when a reaction takes place. Parameter values are 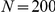, 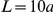, 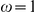, 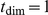, 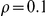.(MPG)
