# Supplementary material for: Confining Domains Lead to Reaction Bursts: Reaction Kinetics in the Plasma Membrane
Source: PLoS One. 2012 Mar 27;7(3):e32948. doi: 10.1371/journal.pone.0032948 (PMC3314009; doi:10.1371/journal.pone.0032948)
Supplement: Text S1 — Supporting information that includes detailed derivations of the mathematical results presented in the main text. (PDF) [file pone.0032948.s005.pdf]

Supporting Text

Confining domains lead to reaction bursts: reaction kinetics in the plasma membrane

Z. Kalay[1], T. K. Fujiwara[1], and A. Kusumi[1,2]

[1]*Institute for Integrated Cell-Material Sciences (WPI-iCeMS),*
[2] *Institute for Frontier Medical Sciences,*
Kyoto University, Kyoto, 606-8501, Japan

February 13, 2012

1 Distribution of escape times

One of the key quantities in our model is the time it takes for one of the molecules to escape from a compartment given they were produced as a result of a dissociation at $t = 0$. Let us denote this random time by t_{e}. If the escape time for the two molecules are represented by the random numbers $t_{1,\mathrm{e}}$ and $t_{2,\mathrm{e}}$ then we have $t_{\mathrm{e}} = \min(t_{1,\mathrm{e}}, t_{2,\mathrm{e}})$. In this section we show that the escape time distribution, f_{e}, is given by a sum of exponentials that can be well approximated by a single exponential under strong confinement. The minimum of two exponentially distributed random numbers is again exponentially distributed whose parameter is the sum of those of the original distributions.

We formulate the problem as a standard first-passage problem as follows. Consider a Brownian particle in a compartment, a square region with permeable boundaries, and calculate the distribution of the time of first exit. We assume that diffusion along x and y directions are independent, which allows us to solve the problem for a one-dimensional (1D) system, and then generalize the results to the two-dimensional (2D) case. Suppose that the molecule is initially placed at $x = x_0$ between two boundaries located at $x = 0$ and $x = L$. When the molecule attempts to cross one of the boundaries, it is sometimes reflected, so that the boundaries can be thought as partially permeable barriers. Once the molecule crosses the boundary it can never go back in. Therefore, the time it leaves the region is always the first exit time. The probability distribution of such a molecule is governed by the diffusion equation

$$\frac{\partial \rho}{\partial t} = D \frac{\partial^2 \rho}{\partial x^2}, \tag{1}$$

with the boundary conditions

$$\begin{aligned} -D \left. \frac{\partial \rho(x,t)}{\partial x} \right|_{x=0} &= -p\rho(0,t), \\ -D \left. \frac{\partial \rho(x,t)}{\partial x} \right|_{x=L} &= p\rho(L,t), \end{aligned} \tag{2}$$

where D is the diffusion constant, and p is a constant related to the permeability of the boundaries such that $p \to 0$, and $p \to \infty$ correspond to impenetrable, and completely permeable boundaries, respectively. The

solution can easily be obtained by using separation of variables [1], and is given in the following form

$$\begin{aligned} \rho(x,t) &= \sum_{n=1}^{\infty} \beta_n(x_0) e^{-\lambda_n^2 Dt/L^2} \phi_n(x), \\ \phi_n(x) &= \gamma_n \left(\alpha\lambda_n \cos(\lambda_n x/L) + \sin(\lambda_n x/L)\right), \\ \gamma_n &= \sqrt{\frac{2}{L}} \left((1+\alpha+\alpha^2\lambda_n^2) + \alpha \left[\frac{\alpha^2\lambda_n^2+1}{\alpha^2\lambda_n^2-1}\right]^2 \cos^2\lambda_n \right)^{-1/2}, \end{aligned} \tag{3}$$

where $\beta_n(x_0)$'s are determined by the initial condition, $\alpha = D/pL$, and λ_n's, $n = 1, 2, 3, ...$, are the positive solutions of

$$\tan\lambda_n = -\frac{2\alpha\lambda_n}{1-(\alpha\lambda_n)^2}. \tag{4}$$

If the molecule is initially at $x = x_0$ such that $\rho(x,t) = \delta(x - x_0)$, the cumulative probability distribution becomes

$$\begin{aligned} \rho_{\text{cdf}}(x,t) &= \int_0^x dy \rho(y,t) \\ &= \sum_{n=1}^{\infty} e^{-\lambda_n^2 Dt/L^2} \frac{\gamma_n L}{\lambda_n} \left(1 - [\cos(\lambda_n x/L) - \alpha\lambda_n \sin(\lambda_n x/L)]\right) \phi_n(x_0), \end{aligned} \tag{5}$$

and finally the distribution of exit times and its cumulative can be related to the above result by [2]

$$\begin{aligned} f_{1,\text{e}}(t) &= -\frac{d}{dt}\rho_{\text{cdf}}(L,t), \\ F_{1,\text{e}}(t) &= \int_0^t ds f_{1,\text{e}}(s) = 1 - \rho_{\text{cdf}}(L,t), \end{aligned} \tag{6}$$

where $f_{1,\text{e}}(t)$ is the escape time distribution for a single molecule, and $F_{1,\text{e}}(t)$ its cumulative.

Now we need to generalize this result to two-dimensions, and obtain the distribution of exit times from a square compartment of area L^2. This could be readily achieved by realizing that the time of first exit is the minimum of the first exit time in x and y directions. Using the well-known result for the distribution of the minimum of two independent random variables [3], we obtain the cumulative probability distribution for the exit time distribution as

$$F_{1,\text{e}}(t) = F_{1,\text{e,x}}(t) + F_{1,\text{e,y}}(t) - F_{1,\text{e,x}}(t)F_{1,\text{e,y}}(t), \tag{7}$$

where $F_{1,\text{e,z}}(t)$ stands for the probability that the molecule exits through one of the boundaries along the z direction, and is explicitly given by

$$F_{1,\text{e,z}}(t) = 1 - \sum_{n=1}^{\infty} e^{-\lambda_n^2 Dt/L^2} \frac{\gamma_n L}{\lambda_n} \phi_n(z_0) \left(1 - [\cos\lambda_n - \alpha\lambda_n \sin\lambda_n]\right), \tag{8}$$

where z_0 is the initial position along the z axis. Usually, the initial condition is not accessible, so that it is reasonable to average over all different initial states. Averaging $F_{1,\text{e,z}}(t)$ over all initial positions z_0 between 0 and L, we get

$$\begin{aligned} \bar{F}_{1,\text{e,z}}(t) &= 1 - \sum_{n=1}^{\infty} e^{-\lambda_n^2 Dt/L^2} \xi_n, \\ \xi_n &= \frac{\gamma_n^2 L}{\lambda_n^2} \left[1 - (\cos\lambda_n - \alpha\lambda_n \sin\lambda_n)\right]^2. \end{aligned} \tag{9}$$

In this case Eq. (7) further simplifies to

$$\bar{F}_{1,\mathrm{e}}(t) = \bar{F}_{1,\mathrm{e,z}}(t)\left(2 - \bar{F}_{1,\mathrm{e,z}}(t)\right),$$
$$\bar{F}_{1,\mathrm{e}}(t) = 1 - \left[\sum_{n=1}^{\infty} e^{-\lambda_n^2 Dt/L^2}\xi_n\right]^2, \tag{10}$$

as there is no difference in the statistics of position between the x and y directions after averaging over the initial position. The probability distribution for the exit times, or the residency time distribution, is given by the first derivative of the last result with respect to time

$$\bar{f}_{1,\mathrm{e}}(t) = 2\sum_{n=1}^{\infty}\sum_{m=1}^{\infty} e^{-(\eta_n+\eta_m)t}\xi_n\xi_m\eta_m \tag{11}$$

where $\eta_n = \lambda_n^2 D/L^2$, and note that $\xi_n\xi_m\eta_m$'s are always positive. The mean and variance of exit time can be calculated from the first two moments of $\bar{f}_{1,\mathrm{e}}(t)$, and are given by

$$\mu_{1,\mathrm{e}} = 2\sum_{n=1}^{\infty}\sum_{m=1}^{\infty}\frac{\xi_n\xi_m\eta_m}{(\eta_n^2+\eta_m^2)^2} \tag{12}$$
$$\sigma_{1,\mathrm{e}}^2 = 4\sum_{n=1}^{\infty}\sum_{m=1}^{\infty}\frac{\xi_n\xi_m\eta_m}{(\eta_n^2+\eta_m^2)^3} - \mu_{\mathrm{e}}^2 \tag{13}$$

For $\alpha = 0$, i.e. no confinement, the summand becomes

$$\frac{\xi_n\xi_m\eta_m}{(\eta_n^2+\eta_m^2)^k} = \frac{L^{2(k-1)}}{D^{k-1}}\frac{4(1-(-1)^n)(1-(-1)^m)}{n^2\pi^2(n^2\pi^2+m^2\pi^2)^k}. \tag{14}$$

In this case, the mean and variance are given by

$$\mu_{1,0,\mathrm{e}} = \frac{2L^2}{D}c_1, \qquad \sigma_{1,0,\mathrm{e}}^2 = \frac{4L^4}{D^2}c_2, \tag{15}$$

where $c_1 = 1.7572\times10^{-2}$, and $c_2 = 5.4247\times10^{-4}$, up to 5 significant digits, and the subscript 0 indicates that there is no confinement.

The contribution from higher order terms in Eq. (11) quickly gets smaller and smaller due to the exponential term. In many cases of practical interest, the first term with $n = 1,\ m = 1$ alone could be a good approximation to the result. To assess the validity of this argument, let us consider the ratio of the exponential factors in the first two terms of the double summation in Eq. (11). This ratio is equal to

$$e^{-(\lambda_2^2-\lambda_1^2)Dt/L^2}. \tag{16}$$

Inspecting the equation for λ_n's, Eq. (4), we notice that $\lambda_{n+1} \approx \lambda_n + \pi$, such that

$$e^{-(\lambda_2^2-\lambda_1^2)Dt/L^2} \approx \left(e^{-Dt/L^2}\right)^{\pi^2+2\lambda_1\pi}, \tag{17}$$

Similarly, the coefficient of the exponential in Eq. (11), given by

$$\xi_n\xi_m\eta_m = \frac{D}{L^2\lambda_n^2}\frac{2\left(1+\zeta_m\cos\lambda_m\right)^2}{(1+\alpha+\alpha^2\lambda_m^2)+\alpha\zeta_m^2\cos^2\lambda_m}\frac{2\left(1+\zeta_n\cos\lambda_n\right)^2}{(1+\alpha+\alpha^2\lambda_n^2)+\alpha\zeta_n^2\cos^2\lambda_n}, \tag{18}$$
$$\zeta_i = \frac{\alpha^2\lambda_i^2+1}{\alpha^2\lambda_i^2-1}, \tag{19}$$

also decays with increasing n and m.

1.1 Escape time when starting from the edge

We are also interested in calculating the escape time distribution for a molecule that has just jumped into a neighboring domain. In the simplest approximation, the initial condition can be taken such that the molecule is uniformly distributed between $0 < x < L$ and $0 < y < a$, where a is the length of a diffusive displacement. In this case the escape time distribution is given by

$$\bar{f}'_{1,\mathrm{e}}(t) = \sum_{n=1}^{\infty}\sum_{m=1}^{\infty} e^{-(\eta_n+\eta_m)t}\xi_n\xi'_m(\eta_m+\eta_n), \tag{20}$$

where

$$\xi'_m = \frac{\gamma_n^2 L^2}{a\lambda_n^2}\left(1-(\cos\lambda_n - \alpha\lambda_n\sin\lambda_n)\right)\left(1-\cos(a\lambda_m/L)+\alpha\lambda_m\sin(a\lambda_m/L)\right). \tag{21}$$

For strong confinement, $\alpha \gg 1$, only the first few terms in Eq. (20) will contribute significantly. Writing out the first three terms that only contain λ_1 and λ_2, and using the following approximations

$$\begin{aligned}\lambda_1^2 &\simeq 2\alpha^{-1} - \frac{1}{3}\alpha^{-2} + O(\alpha^{-3}),\\ \lambda_2^2 &\simeq \pi^2 + 4\alpha^{-1} - \frac{4}{\pi^2}\alpha^{-2} + O(\alpha^{-3}).\end{aligned} \tag{22}$$

we obtain

$$\begin{aligned}\bar{f}'_{1,\mathrm{e}}(t) \simeq{}& 4D\left(\frac{1}{L^2\alpha} - \frac{2L^2-3aL+2a^2}{6L^4\alpha^2} + O(\alpha^{-5/2})\right)e^{-2D\lambda_1^2 t/L^2}\\ &+ 4D\left(\frac{4(\pi^2-3)\sin(a\pi/L)}{3aL\pi^5\alpha^3} + O(\alpha^{-4})\right)e^{-2D(\lambda_1^2+\lambda_2^2)t/L^2},\end{aligned} \tag{23}$$

1.2 Demonstration of how the effective diffusion coefficient is related to the residency time

If the confinement within a compartment is strong, i.e. $\alpha \gg 1$, the residency time distribution given in Eqs. (11) and (20) can be approximated by a single exponential

$$\bar{f}_{1,\mathrm{e}}(t) \approx \frac{1}{\bar{\tau}}e^{-t/\bar{\tau}}, \tag{24}$$

$$\frac{1}{\bar{\tau}} = \frac{2D\lambda_1^2}{L^2}, \tag{25}$$

as $\xi_n \approx 1$, and $\eta_n = \lambda_n^2 D/L^2$. In the limit $\alpha \gg 1$, the first eigenvalue λ_1 approaches 0 such that $\tan\lambda_1 \approx \lambda_1 + \lambda_1^3/3$, and the equation for λ_1 can be replaced by an approximate form

$$\lambda_1 + \frac{\lambda_1^3}{3} \simeq -\frac{2\alpha\lambda_1}{1-(\alpha\lambda_1)^2}, \tag{26}$$

which gives

$$\lambda_1^2 = -\frac{3}{2} + \frac{1}{2\alpha^2}\left(1+\sqrt{1+6\alpha^2+24\alpha^3+9\alpha^4}\right) \simeq \frac{2}{\alpha} - \frac{1}{3\alpha^2} + \frac{10}{9\alpha^3} + O(\alpha^{-4}). \tag{27}$$

Therefore, the average residency time is approximately equal to

$$\bar{\tau} = \frac{L^2}{2D\left(\frac{2}{\alpha} - \frac{1}{3\alpha^2} + \frac{10}{9\alpha^3} + O(\alpha^{-4})\right)} \approx \frac{L^2}{4\frac{D}{\alpha}}. \tag{28}$$

Although we found a relation between the residency time and other parameters, we still need to take one more step and express α in terms of a quantity that can be measured in experiments. In the strong confinement limit

that we are interested in, a Brownian particle behaves much like a random walker in a 2D lattice with lattice spacing L. In this picture, each lattice site corresponds to a square compartment of area L^2, and the random walker takes steps between adjacent lattice sites at a rate $F = 1/4\bar{\tau}$. The properties of this random walk can be described by using a Master equation and various transport quantities can be readily calculated [4]. With a straightforward calculation, it can be shown that the mean square displacement of a random walker which hops between lattice sites with an average residency time $\bar{\tau}$ is given by

$$\langle x^2 \rangle = 4\frac{L^2}{4\bar{\tau}}t = 4D_{\text{eff}}t, \tag{29}$$

which implies that $D/\alpha \approx D_{\text{eff}}$. The average residency time, and its variance can now be expressed as

$$\mu_{1,\text{e}} = \frac{L^2}{4D_{\text{eff}}}, \quad \sigma^2_{1,\text{e}} = \frac{L^4}{16D^2_{\text{eff}}}, \tag{30}$$

where all quantities are experimentally measurable.

1.3 Two molecules

In the sections above, we obtained the mean and variance of the escape time for a single molecule; however, the quantity of main interest is the time it takes until *any* of the two molecules produced after the dissociation escapes. This is simply the minimum of $t_{1,\text{e}}$ and $t_{2,\text{e}}$ as stated earlier, and the statistics is given by

$$\mu_{\text{e}} = \frac{L^2}{8D_{\text{eff}}}, \quad \sigma^2_{\text{e}} = \frac{L^4}{64D^2_{\text{eff}}}, \tag{31}$$

where we used the fact that $t_{1,\text{e}}$ and $t_{2,\text{e}}$ are both exponentially distributed variables.

The results in Eq. (31) are valid when an average over initial positions is performed. The results relevant for the case where a molecule starts at the edge of the compartment, i.e. just entered the compartment, can be derived from Eq. (23). However, when the confinement effect is strong, the difference between the two cases becomes negligible, as seen from Eq. (23).

2 The relation between α, p, and D_{eff}/D

In order to establish a relation between D_{eff}/D, and p or α, we make use of the Master equation for random walks in a 1D lattice with nearest neighbor transitions, and its equivalence to the diffusion equation in the continuum limit [4]. Consider a 1D lattice with spacing a with sites located at $x_m = a(2m+1)/2$, $m = 0, \pm1, \pm2, ...$, and partitioned into compartments by links with lower transfer rates f at a spatial period of L, as in ref. [5], and as illustrated Fig. S1 below. Let $\rho(x)$ be the probability density of finding the particle in a 1D continuous space such that $P_m = \int_{ma}^{(m+1)a} dx\rho(x)$ is comparable to the probability of finding the particle at the mth lattice site in the Master equation picture. Integrating the continuity equation over space, we can relate the parameters in the Master equation to the boundary conditions in the continuum picture, as outlined below. Suppose that the random walker occupies site 0 with probability 1 at $t = 0$. At $t \sim 0$, the rate of change of probability of finding a random walker at site 0 obeys

$$\frac{dP_0}{dt} = -(F+f)P_0 + FP_1. \tag{32}$$

Meanwhile, the probability density $\rho(x)$ is governed by the diffusion equation

$$\frac{\partial \rho}{\partial t} = -\nabla \cdot \vec{J} = -\frac{\partial}{\partial x}\left(-D\frac{\partial \rho}{\partial x}\right), \tag{33}$$

where $\vec{J}$ is the probability flux. Therefore, we have the following relation between P_0 and the probability flux at the boundaries of the region $[0, a]$

$$\frac{dP_0}{dt} = \int_0^a dx \frac{\partial \rho}{\partial t} = D\left.\frac{\partial \rho}{\partial x}\right|_{x=a} - D\left.\frac{\partial \rho}{\partial x}\right|_{x=0}. \tag{34}$$

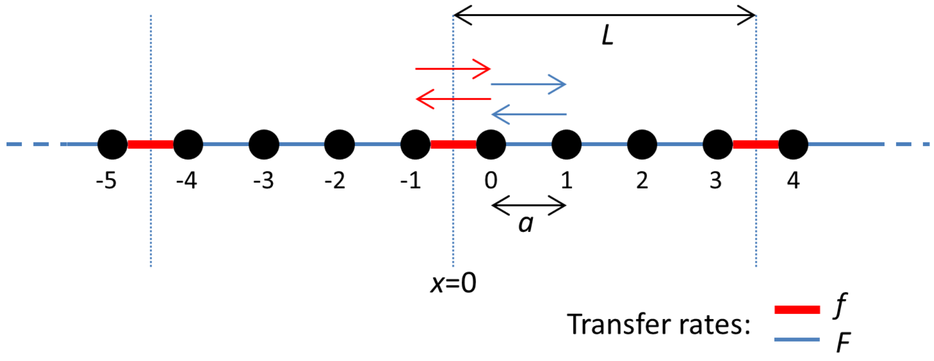

Figure S1: **Illustration of the Master equation picture of diffusion with periodically placed barriers.**

Combining the last result with Eq. (32), we obtain

$$D \left.\frac{\partial \rho}{\partial x}\right|_{x=a} - D \left.\frac{\partial \rho}{\partial x}\right|_{x=0} = -(F+f)P_0 \tag{35}$$

As the transfer rate F and f are associated with escaping from the boundaries at $x = a$, and $x = 0$, respectively, we deduce

$$-D \left.\frac{\partial \rho}{\partial x}\right|_{x=a} = F P_0, \quad -D \left.\frac{\partial \rho}{\partial x}\right|_{x=0} = -f P_0. \tag{36}$$

Furthermore, we can make use of the approximation $\rho(0) \simeq P_0/a$, as $a \to 0$, to write

$$-D \left.\frac{\partial \rho}{\partial x}\right|_{x=a} = F a \rho(0), \tag{37}$$

$$-D \left.\frac{\partial \rho}{\partial x}\right|_{x=0} = -f a \rho(0). \tag{38}$$

Comparing Eq. (38) with the boundary condition we gave in Eq. (2), we get

$$p = f a. \tag{39}$$

For a completely reflecting boundary $p = 0$ such that $f = 0$, and for a completely absorbing boundary, we have $p \to \infty$. It was previously shown that in a lattice partitioned into compartments of length L between which the transfer rate is equal to f with all other transfer rates being F, transport at long times can be characterized by an effective transfer rate [5]

$$F_{\text{eff}} = \frac{f}{a/L + (f/F)(1 - a/L)}, \tag{40}$$

so that the mean square displacement at times longer than the escape time from a compartment goes as $\langle x^2 \rangle = 2F_{\text{eff}} a^2 t$, where a is the lattice spacing. Rearranging Eq. (40), and using Eq. (39) we get

$$p = \frac{F_{\text{eff}} a^2}{L\left[1 - (F_{\text{eff}}/F)(1 - a/L)\right]}, \tag{41}$$

which relates p to observable quantities. Note that in the continuum limit, i.e. $a \to 0$, the effective hopping rate given in Eq. (40) becomes the effective diffusion coefficient

$$\lim_{a \to 0} F_{\text{eff}} a^2 = D_{\text{eff}}, \tag{42}$$

such that p is given by

$$p = \frac{D_{\text{eff}}}{L\left(1 - D_{\text{eff}}/D\right)}. \tag{43}$$

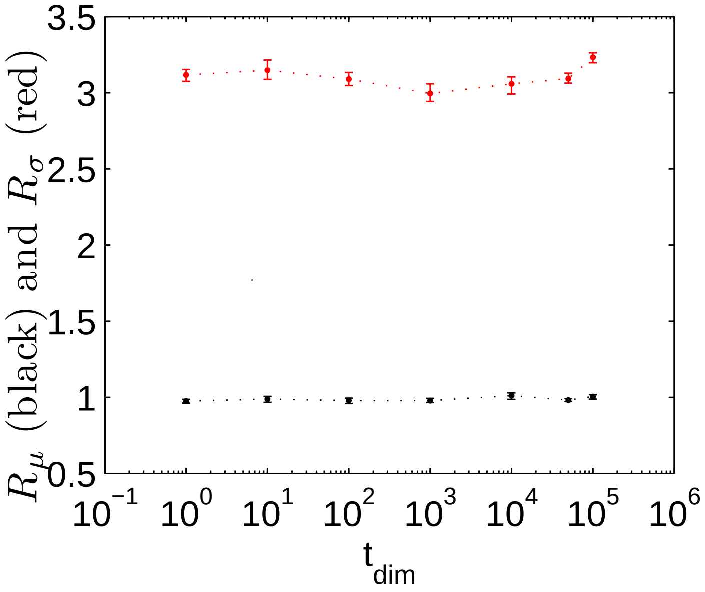

Figure S2: **Behavior of R_μ and R_σ (described in the text) as a function of t_{dim}.** Data obtained by Monte Carlo simulations. Parameters values are $P_{\text{cross}} = 9 \times 10^{-4}$, $N = 200$, $L = 20a$, $\rho = 0.1$. Error bars were obtained by subsampling the data by bootstrapping and indicate 95% confidence intervals.

Finally, we find that the effective diffusion coefficient is related to p and $alpha$ by

$$D_{\text{eff}} = \frac{D}{1 + D/pL} = \frac{D}{1 + \alpha}, \tag{44}$$

where we used the relation $\alpha = D/pL$ (see Introduction section of the main text).

3 The effect of dimer lifetime

In the main text, we did not present simulation results for different values of dimer lifetime. As shown here in Figure S2, we found that the effect of dimer lifetime on the mean and variance is the same whether confining domains are present or not.

4 Ensemble averaged reaction rate

Here we present the effect of ensemble averaging on the data shown in Figure 1(a). We obtained 1000 data sets for four different confinement strengths, $P_{\text{cross}} = 2.4 \times 10^{-4},\ 9.8 \times 10^{-4},\ 7.9 \times 10^{-3},\ 2.5 \times 10^{-1}$, each containing 1000 reaction events in time. Representative single data sets corresponding to each confinement strength is displayed in Figure 1(a). After ensemble averaging over 1000 data sets, we found that the reaction rate is the same no matter what the confinement strength is, at all times. This is expected as the random process that governs the encounter of molecules is a stationary process, making the ensemble average identical to the time average. Figure S3 contains the plots of ensemble averaged number of reactions versus time whose slopes, i.e. reaction rates, do not depend on confinement strength.

5 Partition function for a system reversibly reacting particles

Consider a system of N particles undergoing the reaction $M + M \leftrightarrow D$ such that the number of monomers (M) and dimers (D) at equilibrium are given by N_{M} and $N_{\text{D}} = (N - N_{\text{M}})/2$, respectively.The energy of this system can be expressed as the sum of the energies of monomers and dimers, $E_{\text{M}}(L, \alpha)$ and $E_{\text{D}}(L, \alpha)$, and the interaction energy between them $E_{\text{I}}(N_{\text{M}}, N_{\text{D}}, L, \alpha)$. All of these energies may depend on L, the characteristic

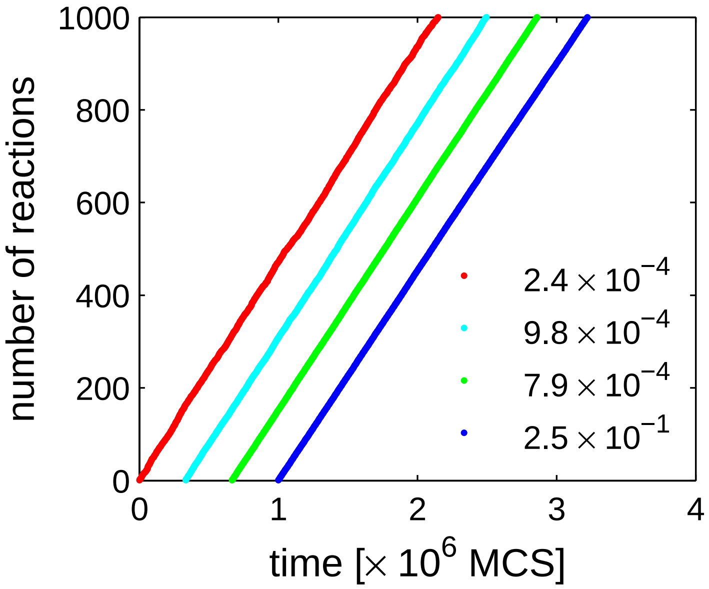

Figure S3: **Total number of reactions involving the tracer molecule as a function of time**. Results are obtained by Monte Carlo simulations (ensemble averaged over 1000 non-overlapping segments of the simulation data). All definitions and parameter values are the same as those of Figure 1(a) of the main text. For visual clarity, each data set is plotted with a different offset along the x-axis.

length of confinement, α, the confinement strength, and other properties of the environment which are not explicitly considered in this discussion. The partition function [6] for this system can be written as

$$Z = \sum_{k=0}^{N/2} \binom{N}{2k} e^{-\beta[NE_{\mathrm{D}}(L,\alpha)/2+2k\Delta E(L,\alpha)+E_{\mathrm{I}}(k,L,\alpha)]}, \tag{45}$$

where $\Delta E = E_{\mathrm{M}}(L,\alpha) - E_{\mathrm{D}}(L,\alpha)/2$, $\beta = 1/k_{\mathrm{B}}T$, k_{B} is the Boltzmann constant, T is the absolute temperature, and the index k corresponds to the number of pairs of monomers. If the interaction energy E_{I}, is negligible, and E_{M} and E_{D} do not depend on L and α the partition function can be approximated for large values of N by

$$\begin{aligned} Z &= \sum_{k=0}^{N/2} \binom{N}{2k} e^{-\beta[NE_{\mathrm{D}}/2+2k\Delta E]} \simeq \frac{1}{2} e^{-\beta NE_{\mathrm{D}}/2} \sum_{k=0}^{N} \binom{N}{k} e^{-\beta k\Delta E}, \\ &\simeq \frac{1}{2} e^{-\beta NE_{\mathrm{D}}/2} \left(1 + e^{-\beta\Delta E}\right)^{N}, \end{aligned} \tag{46}$$

such that the average energy per particle is given by

$$\frac{\langle E \rangle}{N} = -\frac{1}{N}\frac{\partial}{\partial\beta} \ln Z \simeq \frac{E_{\mathrm{D}}}{2} + \Delta E \frac{e^{-\beta\Delta E}}{1 + e^{-\beta\Delta E}}. \tag{47}$$

Note that the average energy per particle can also be written as

$$\frac{\langle E \rangle}{N} = \frac{N_{\mathrm{M}}E_{\mathrm{M}}}{N} + \frac{N_{\mathrm{D}}E_{\mathrm{D}}}{N}. \tag{48}$$

Setting Eq. (47) and Eq. (48) equal and solving for N_{M}/N, we obtain

$$\frac{N_{\mathrm{M}}}{N} \simeq \frac{1}{1 + e^{\beta(E_{\mathrm{M}} - E_{\mathrm{D}}/2)}}, \tag{49}$$

which is equal to $1/2$ as $T \to \infty$, becomes 0 as $T \to 0$ if the dimer state is favorable ($E_{\mathrm{M}} - E_{\mathrm{D}}/2 > 0$), and becomes 1 as $T \to 0$ in case the monomer state is favorable ($E_{\mathrm{M}} - E_{\mathrm{D}}/2 < 0$).

6 Statistics of burst amplitude and the derivation of P_{fug}

When two molecules encounter, the number of times they will encounter again depends on how long they will stay together in that compartment. If the encounter time happens to be larger than the escape time, then one of the molecules will leave, leading to a significant increase in the time it takes before the next collision takes place. The statistics of this occurrence can be described by a random variable which is the difference of the escape time and the encounter time. Let $t_{\text{d}} = t_{\text{r}} - t_{\text{e}}$ be the random variable that is positive when one of the molecules leave the domain before an encounter and negative otherwise, where t_{r} is the *re*-encounter time with the corresponding distribution $f_{\text{r}}(t)$. We are interested in calculating how often t_{d} is positive.

$$f_{\text{d}}(t_{\text{d}}) = \int_0^\infty dt_{\text{e}} f_{\text{e}}(t_{\text{e}}) f_{\text{r}}(t_{\text{e}} + t_{\text{d}}), \tag{50}$$

as the distribution of the difference of two random variables is their cross correlation. The integral in Eq. (50) needs to be separated into two parts, in which t_{d} has different signs

$$f_{\text{d}}(t_{\text{d}}) = \begin{cases} \displaystyle\int_0^\infty dt_{\text{e}} f_{\text{e}}(t_{\text{e}}) f_{\text{r}}(t_{\text{e}} + t_{\text{d}}), & \text{when } t_{\text{d}} > 0, \\ \displaystyle\int_{-t_{\text{d}}}^\infty dt_{\text{e}} f_{\text{e}}(t_{\text{e}}) f_{\text{r}}(t_{\text{e}} + t_{\text{d}}), & \text{when } t_{\text{d}} < 0. \end{cases} \tag{51}$$

Therefore, after the two molecules separate from an encounter, the probability that one of them will leave before the next collision is

$$P_{\text{fug}} = \int_0^\infty dt_{\text{d}} \int_0^\infty dt_{\text{e}} f_{\text{e}}(t_{\text{e}}) f_{\text{r}}(t_{\text{e}} + t_{\text{d}}) = \lim_{\epsilon \to 0} \widetilde{f}_{\text{d},t_{\text{d}}>0}(\epsilon), \tag{52}$$

where the tilde denotes Laplace transform as usual. This reduces to

$$P_{\text{fug}} = \lim_{\epsilon \to 0} \int_0^\infty dt_{\text{e}} f_{\text{e}}(t_{\text{e}}) e^{\epsilon t_{\text{e}}} \left(\widetilde{f}_{\text{r}}(\epsilon) - \int_0^{t_{\text{e}}} du f_{\text{r}}(u) \right) \tag{53}$$

$$= \int_0^\infty dt_{\text{e}} F_{\text{e}}(t_{\text{e}}) f_{\text{r}}(t_{\text{e}}), \tag{54}$$

where $F_{\text{e}}(t_{\text{e}})$ is the cumulative of the escape time distribution. If the escape time distribution is a sum of exponentials, such that

$$F_{\text{e}}(t_{\text{e}}) = 1 - \sum_{i=1}^\infty \frac{\alpha_i}{\beta_i} e^{-\beta_i t_{\text{e}}}, \tag{55}$$

the expression for P_{fug} simplifies to

$$P_{\text{fug}} = 1 - \sum_{i=1}^\infty \frac{\alpha_i}{\beta_i} \lim_{\epsilon \to 0} \widetilde{f}_{\text{r}}(\epsilon + \beta_i). \tag{56}$$

If $\beta_i \ll 1$, we can use the Taylor expansion of $\widetilde{f}_{\text{r}}(\epsilon)$ around 0, instead of its full form so that

$$\lim_{\epsilon \to 0} \widetilde{f}_{\text{r}}(\epsilon + \beta_i) \simeq 1 + \epsilon \left. \frac{d\widetilde{f}_{\text{r}}(\epsilon)}{d\epsilon} \right|_{\epsilon \to \beta_i} + \frac{\epsilon^2}{2} \left. \frac{d^2 \widetilde{f}_{\text{r}}(\epsilon)}{d\epsilon^2} \right|_{\epsilon \to \beta_i} \tag{57}$$

This results in the notable simplification

$$P_{\text{fug}} \simeq \sum_{i=1}^\infty \alpha_i \left(\mu_{\text{r}} - \frac{\beta_i \left(\sigma_{\text{r}}^2 + \langle t_{\text{r}} \rangle^2 \right)}{2} \right), \tag{58}$$

in which only the mean and variance of the encounter time distribution is involved. When $f_{\text{e}}(t_{\text{e}})$ is well approximated by a single exponential such that $\alpha_1 = \beta_1$, and $\beta_1 = \mu_{\text{e}}^{-1}$,

$$P_{\text{fug}} \simeq 1 - \lim_{\epsilon \to 0} \widetilde{f}_{\text{r}}(\epsilon + \mu_{\text{e}}^{-1}) \simeq \frac{\mu_{\text{r}}}{\mu_{\text{e}}} - \frac{\sigma_{\text{r}}^2 + \mu_{\text{r}}^2}{2\mu_{\text{e}}^2}. \tag{59}$$

We immediately see that if the dimensionless average residency time is much larger than 1, $P_{\text{fug}} \simeq 0$, and if it is much smaller than 1, P_{fug} depends on the short time behavior of $f_{\text{r}}(t_{\text{r}})$.

6.1 Statistics of the amplitude of bursts

Given a pair of molecules dissociated at $t = 0$, the number of times the pair will encounter before one of the molecules escapes will be distributed according to

$$P_{\text{enc}}(n) = P_{\text{fug}}(1 - P_{\text{fug}})^n. \tag{60}$$

Upon an encounter, the particles can react with probability ω. Therefore, the probability that k reactions take place in n encounters is given by

$$P_{\text{rxn}}(k|n) = \binom{n}{k} \omega^k (1-\omega)^{n-k}, \tag{61}$$

which is the Bernoulli distribution. Using Bayes law, summing over all possible values of n, and assuming that the particles dissociated at $t = 0$, we obtain the probability for the occurrence of k more reactions before the particles move into different compartments as

$$P_{\text{rxn}}(k+1) = P_{\text{fug}} \sum_{n=0}^{\infty} \binom{n}{k} \left(\frac{\omega}{1-\omega}\right)^k \left[(1-\omega)(1-P_{\text{fug}})\right]^n, \tag{62}$$

such that $P_{\text{rxn}}(m)$ is the probability of having m reactions in a burst, where $m = 1, 2, 3, .$ The mean number of reactions in a burst and its variance are readily calculated to be

$$\mu_{\text{amp}} = 1 + \omega \frac{1 - P_{\text{fug}}}{P_{\text{fug}}}, \quad \sigma^2_{\text{amp}} = \mu_{\text{amp}}(\mu_{\text{amp}} - 1) \tag{63}$$

An approximation for P_{fug} as a function of all the relevant physical parameters in the system is given in Eq. (41). Note that the constant 1 that appears in μ_{amp} reflects the fact that this calculation is valid when the particles certainly react after entering the same compartment.

7 Distribution of the time between reactions

In the main text, our analysis of the simulation data was largely constricted to calculating the mean and variance of the time between reaction events. Without doubt, the behavior of the full distribution of the time between reactions is richer. Below, we plot this distribution for various confinement strengths, with the same parameter values as in Fig 3(a) of the main text. An obvious feature of the curves in Fig. S4 is that, as the confinement effect gets stronger (with decreasing P_{cross}), the probability of having the next reaction sooner becomes higher. In the absence of an array of confining domains, this distribution is characterized by a slowly decaying function, regime 1 (reg. 1), followed by an exponential decay, regime 2, as illustrated by the dark blue arrows in Fig. S4(B). The presence of confining domains leads to the emergence of an additional regime, reg. 2 (cyan), where the distribution decays slower than an exponential. A detailed mathematical analysis of this distribution for random walks in lattices as well as in continuum will be presented in a forthcoming publication.

References

[1] H. S. Carslaw and J. C. Jaeger. *Conduction of Heat in Solids.* Clarendon Press, Oxford, 2nd ed edition, 1986.

[2] S. Redner. *A Guide to First-Passage Processes.* Cambridge University Press, Cambridge, UK, 2001.

[3] E. J. Gumbel. *Statistics of Extremes.* Dover Publications, Mineola, N.Y, dover edition edition, 2004.

[4] B. D Hughes. *Random Walks and Random Environments.* Clarendon Press, Oxford, 1995.

[5] V. M. Kenkre, L. Giuggioli, and Z. Kalay. Molecular motion in cell membranes: Analytic study of fence-hindered random walks. *Phys Rev E*, 77(5):051907, May 2008.

[6] L. D Landau and E. M Lifshitz. *Statistical Physics, Third Edition, Part 1: Volume 5.* Butterworth-Heinemann, 3 edition, January 1980.

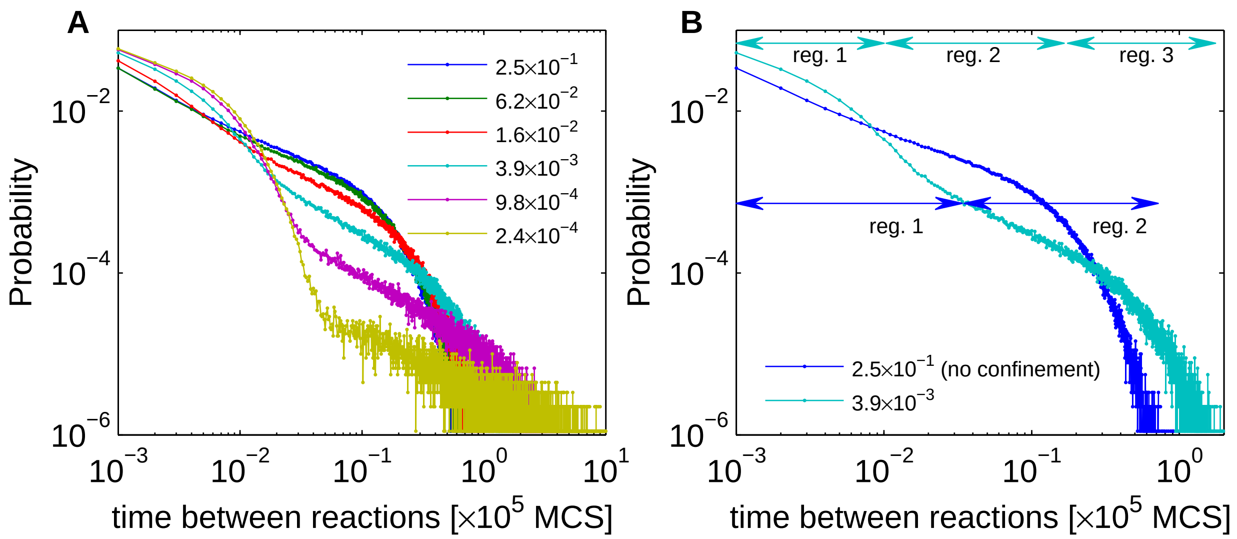

Figure S4: **Distribution of the time between reactions for different confinement strengths**. **A.** All parameter values are the same as in Fig 3(a) of the main text. A bin size of 100 simulation steps was used to calculate the probabilities. The numbers in the legend correspond to the value of P_{cross}. **B.** distribution of the time between reactions for $P_{\mathrm{cross}} = 2.5 \times 10^{-1}$ (no confinement) and 3.9×10^{-3}, illustrating the qualitative change in the behavior of the curves in different time windows. All parameter values are the same as those in **A**. See text for further details.
